# They Are Like Family: A Qualitative Thematic Analysis of Nurses’ Experiences in a Tshwane Dialysis Unit

**DOI:** 10.3390/healthcare14050622

**Published:** 2026-02-28

**Authors:** Morakane Audrey Mphokela, Jacobeth Malesela, Moreoagae Bertha Randa

**Affiliations:** 1 Department of Nursing Science, Sefako Makgatho Health Sciences University, Pretoria 0208, South Africa; 202220014@swave.smu.ac.za (M.A.M.); jackie.malesela@gmail.com (J.M.); 2 Department of Public Health, Sefako Makgatho Health Sciences University, Pretoria 0208, South Africa

**Keywords:** chronic kidney disease, renal dialysis, nurses, nursing care, qualitative research, nephrology

## Abstract

**Highlights:**

**What are the main findings?**

- Dialysis nurses develop deep, family-like relationships with patients, which provide meaning but also expose them to emotional exhaustion, moral distress, and compassion fatigue.
- Persistent shortages of human and material resources along with limited managerial visibility and support significantly hinder nurses’ ability to deliver safe, compassionate, and holistic Chronic kidney disease care.

**What are the implications of the main findings?**

- Strengthening leadership presence, emotional support systems, and nephrology-focused staff development is essential to sustaining nurse wellbeing and improving the consistency and quality of dialysis care.
- Addressing systemic gaps in staffing, equipment availability, and timely maintenance is critical for reducing moral distress, preventing avoidable patient harm, and advancing equitable CKD care aligned with Sustainable Development Goals 3.

**Abstract:**

**Background**: Chronic kidney disease (CKD) continues to place immense strain on health systems globally, with nurses at the centre of care delivery physically, emotionally, and relationally. In dialysis units, nurses form long-term therapeutic relationships with patients who depend on life-sustaining treatment several times a week. **Objective**: This study explored the lived experiences of professional nurses caring for patients with CKD in a dialysis unit, using Watson’s Theory of Human Caring as a guiding framework. **Methods**: A qualitative, exploratory, descriptive design was employed. Data were collected through in-depth face-to-face interviews with twelve professional nurses and analyzed using thematic analysis. Trustworthiness was ensured through credibility, dependability, confirmability, transferability, and authenticity. Ethical approval and informed consent were obtained. **Results**: Three themes emerged: (1) emotional and professional experiences, (2) systemic resource constraints, and (3) recommendations for practice improvement. These findings highlight the tension between caring ideals and systemic limitations. **Conclusions**: The study concludes that dialysis nursing is profoundly meaningful yet emotionally demanding. Strengthened emotional support, improved leadership visibility, consistent resource allocation, and enhanced nephrology nursing education are critical to sustaining compassionate care. The findings offer important insights for policy, workforce development, and quality improvement efforts in CKD care.

## 1. Introduction

Chronic kidney disease (CKD) represents a significant and escalating global health concern, affecting more than 850 million people and contributing significantly to morbidity, mortality, and long-term disability [1]. Recent analyses indicate that its prevalence continues to rise due to ageing populations, increased rates of hypertension and diabetes, and often late diagnoses, particularly in low- and middle-income countries [2,3]. In South Africa, CKD care is further complicated by health system inequalities, shortages of specialized personnel, and limited access to renal replacement therapy [4]. As a result, dialysis units frequently operate under immense pressure, making nurses central to both the technical and relational components of CKD management.

Dialysis nursing requires a delicate interplay between advanced clinical skills and deep emotional engagement. Nurses are tasked with managing complex procedures, monitoring fragile patients, and providing continuous psychosocial support to individuals undergoing lifelong treatment [5]. The close therapeutic relationships built over months or years are both meaningful and emotionally demanding, as nurses witness patient suffering, deterioration, and mortality [6]. International literature consistently highlights elevated levels of burnout, moral distress, and compassion fatigue among dialysis nurses, exacerbated by understaffing, resource constraints, and heavy workloads [7,8,9,10].

It is critical to distinguish between chronic kidney disease (CKD) and acute kidney injury (AKI), as these conditions follow distinct clinical trajectories with differing emotional and care implications. CKD is characterized by a progressive and often irreversible decline in kidney function, requiring long-term renal replacement therapies such as dialysis that may continue for many years [11]. AKI, in contrast, involves a sudden deterioration in kidney function that is frequently reversible with timely and appropriate intervention, allowing some patients to recover fully and discontinue dialysis [12]. Nurses caring for patients with AKI have reported more positive emotional experiences, including hope and relief associated with patient recovery and treatment cessation [13]. Conversely, caring for patients with CKD, particularly those with end-stage renal disease, involves sustained emotional labour linked to disease chronicity, uncertain prognosis, and, in many cases, the terminal nature of the condition [14]. Recognizing this contrast is critical for comprehending the emotional terrain of dialysis nursing, which entails negotiating both the persistent burden of chronic sickness and the brief times of patient recovery.

The global and local burden of CKD highlights the vital necessity of Sustainable Development Goals (SDG) 3, which seeks to reduce mortality from noncommunicable diseases (NCDs) and attain universal health coverage [15]. CKD is a major health concern, fueled by ageing populations, lifestyle choices, and systemic inequities, particularly in resource-constrained countries like South Africa [16,17]. To address this difficulty, a compassionate and holistic strategy based on caring science is necessary, in addition to good clinical management.

In addition to providing technical treatments, nurses offer emotional and psychosocial support. However, research shows that systemic pressures lead to high levels of burnout, moral distress, and compassion fatigue among dialysis nurses [18].

Despite the extensive study of clinical and systemic challenges of CKD in South Africa, significant gaps persist in the body of knowledge. There is notable lack of in-depth investigation into nurses’ actual lived experiences in managing CKD, particularly within resource limited settings. For example, while a qualitative study explored compassion fatigue among dialysis nurses [8] a more thorough investigation is required to comprehend how systemic issues like resource scarcity and emotional strain specifically affect nurses’ personal and professional lives in this context.

Notably, there is underutilization of contextualized theoretical frameworks such as Watson’s Theory of Human Caring, to examine the relational and emotional facets of CKD care which are central to holistic practice [19].

Quality and holistic care for patients living with CKD is closely aligned with SDG 3, which aims to promote good health and wellbeing through equitable access to quality health services and the reduction in non-communicable disease burden [15]. For individuals dependent on long-term dialysis, achieving these goals requires more than effective clinical management and access to treatment; it also depends on compassionate, person-centred care that attends to their emotional and psychosocial needs.

This study is guided by Watson’s Theory of Human Caring, which conceptualizes nursing as a relational, ethical, and human-centred practice sustained through authentic presence, transpersonal relationships, and supportive caring-healing environments. In resource-constrained dialysis settings, this framework is particularly relevant for understanding how systemic and organizational conditions shape nurses’ ability to deliver compassionate and equitable care. These concerns align directly with SDG 3, which emphasizes universal health coverage, quality healthcare services, and the reduction in non-communicable disease burden. Situating nurses lived experiences within these frameworks enables a coherent examination of care quality, workforce wellbeing, and health equity in CKD services.

The primary aim of this research is to explore nurses’ lived experiences in a dialysis unit, situating these experiences within broader health equity and quality-of-care objectives.

## 2. Materials and Methods

### 2.1. Study Design

This study adopted a qualitative, exploratory, descriptive design to gain in-depth insights into the experiences of nurses caring for CKD patients in an academic hospital in Tshwane, Gauteng. From an ethical perspective, the qualitative design was appropriate as it prioritized participant autonomy, enabled control over the depth of disclosure, and avoided invasive data collection methods. This approach allowed nurses to narrate experiences at their own pace, thereby reducing emotional risk and supporting psychological safety.

### 2.2. Study Context

The clinical setting for this study is a dialysis unit at an academic hospital, Gauteng Province in South Africa, which treats 70 CKD patients and 20 AKI patients requiring haemodialysis annually. The health team comprises 21 professional nurses, two enrolled nurses and one auxiliary nurse and a nephrologist who all work collaboratively to provide patient care within a resource constrained environment The selection of this facility was justified by the volume of clinical activity; specifically, an average of 30 chronic-regime patients were attended to daily, assuring a robust data pool and making the site highly suitable for intensive data collection. The research team assessed risks of indirect identification and mitigated these through de-identification strategies, aggregation of contextual data, and the removal of specific organizational markers.

All identifying institutional characteristics were deliberately limited to protect participant anonymity. The potential risks of indirect identification were assessed, and mitigation strategies included data de-identification, aggregation of contextual information, and the removal of specific organizational markers.

### 2.3. Population and Sampling

A total of 19 professional nurses were purposively recruited. Data saturation was reached by the twelve interviews, at which point recruitment was discontinued as the data was sufficient to address the study objectives [20]. Inclusion criteria required participants to have a year and more experience in dialysis nursing to ensure that they had adequate exposure to the clinical and emotional demands associated with CKD care. Furthermore, participants were required to be categorized as either specialty-trained (holding a post-basic qualification in Nephrology) or non-specialty professional nurses, and all provided signed informed consent indicating their willingness to participate. Exclusion criteria included nurses who had less than a year’s experience, as well as those in direct supervisory or managerial relationships with the researchers. To mitigate perceived coercion, recruitment was conducted independently of managerial structures, participation was voluntary, and assurances were provided that non-participation would have no consequences. Measures were put in place to ensure referrals of participants who experienced emotional distress during the interview.

### 2.4. Recruitment Strategy

Following ethical approval, the researcher met with the dialysis unit manager and explained the study’s purpose. Subsequently, nurses meeting the inclusion criteria and interested to participate in the study were briefed about the study’s objective, the process to unfold; and were also given an opportunity to ask questions by the researcher. Consent to the interviews, and to the use of an audiotape were obtained prior to participation.

### 2.5. Data Collection

Data were collected through in-depth, semi-structured interviews, allowing for deep exploration of nurses’ emotional, relational, and professional experiences. Individual interviews were scheduled based on participant availability, minimizing disruption and stress. Interviews were conducted in a private staff meeting room within the workplace to ensure accessibility while minimizing participant burden. The setting safeguarded confidentiality and privacy and supported emotional safety by allowing participants to engage in a familiar environment. The nurses were given the option to pause or discontinue the interview if they experienced discomfort.

The interview guide included open-ended questions designed to explore nurses’ experiences, relational dynamics with patients, and perceptions of organizational support. It is worth noting that the interview guide was piloted with two participants from the same setting who did not form part of the main study. Only one question required modification, which was adjusted based on the nurses’ feedback to ensure adequate coverage of the research objectives. Data were collected between April and May 2024. The central question posed was: “What is your experience as a nurse working in the renal unit caring for patients with chronic kidney disease?” Probes were used based on participants’ responses. The interviews, conducted in English, lasted approximately 40 to 60 min and were audio-recorded with participant permission, while being supplemented with field notes. Data saturation was reached by the twelve interviews, at which point recruitment was discontinued, as the data was sufficient to address the study objectives.

### 2.6. Data Analysis

Data were analyzed using thematic analysis following Datt and Chetty’s approach [21]. All interview recordings were transcribed verbatim, after which the transcripts were read repeatedly to develop familiarity with the data. Initial open coding was then undertaken to identify meaningful units of text. Coding was conducted inductively to allow themes to emerge from the data, while also being informed by the study objectives and Watson’s Theory of Human Caring.

NVivo version 14 was used to support the organization and management of the data. Codes were compared and grouped into broader categories, from which preliminary themes were developed and refined through constant comparison across transcripts. An independent coder reviewed the coding process, and consensus discussions were held to finalize the themes. Verbatim quotations were used to support the findings and enhance transparency.

Although the analysis was primarily inductive, Watson’s Theory of Human Caring informed the interpretive phase by sensitizing the researchers to relational, emotional, ethical, and organizational dimensions of caring reflected in nurses’ accounts.

### 2.7. Bracketing and Reflexivity

The researchers understand that their backgrounds and viewpoints may influence data collecting and interpretation. To reduce potential biases, they kept a reflective notebook throughout the study, reflecting on their assumptions and interactions with participants. Furthermore, independent coder and peer debriefings were used to improve objectivity and trustworthiness [22]. The research team maintained sensitivity to the participants’ different experiences, ensuring that the voices of nurses were genuinely represented in the findings.

### 2.8. Trustworthiness

Trustworthiness was ensured through Lincon and Guba’s [22] framework. Credibility was enhanced through prolonged engagement, peer debriefing, member checking, whilst the use of audit trail and consistent documentation ensured dependability. Transferability was ensured through detailed contextual descriptions; and confirmability was enhanced through reflexive journaling and external review.

### 2.9. Ethical Considerations

Ethical clearance was obtained from Sefako Makgatho Health Sciences University Research Ethics Committee (SMUREC/H/30/2024:PG). Permission to conduct the study was granted by the National Health Research Database committee, the Chief Executive Officer (CEO) of the academic hospital, and the manager of the dialysis unit within the academic hospital. Participation was voluntary, written informed consent obtained; and participants were informed of their rights to withdraw from the study at any time. Confidentiality and anonymity were maintained by assigning code numbers [23].

Generative artificial intelligence (GenAI) has been used in this paper to assist to generate the Tables.

## 3. Results

### 3.1. Findings

The nurses that participated in the study (Table 1) were all female. Their ages ranged between 34 and 55 years. The nurses’ highest professional qualifications were diplomas in nursing (general, psychiatric, and community), midwifery, and bachelor’s degrees in nursing. Some nurses had specialized training in nephrology nursing, whereas others did not. Nurses’ experience ranged from one year and three months to 22 years. The years of experience working in the renal unit ranged from less than 12 months to 19 years.

The findings in Table 1 show that the participants were largely experienced professional nurses with varied educational backgrounds and differing levels of specialist training and renal unit experience. This range of experience provided an appropriate context for exploring nurses’ perspectives related to the study focus.

Three overarching themes emerged from the analysis, as presented in Table 2: Emotional and Professional Experiences, Systemic Resource Constraints, and Recommendations for Practice Improvement. Together, these themes reflect the complex emotional, professional, and structural realities of nurses caring for patients with chronic kidney disease in a dialysis unit.

#### 3.1.1. Theme: Emotional and Professional Experiences

This theme captures the emotional demands of dialysis nursing, reflecting both psychological strain and sources of professional fulfilment.

Participants consistently describe emotional exhaustion, frustration, and distress, which they attributed largely to systemic pressures rather than to patient care itself. High workloads, limited staffing, and inadequate managerial support intensified stress and contributed to moral distress when nurses were unable to provide the level of care they valued.

One participant emphasized the importance of leadership presence:

*“If nursing management, particularly the matron, were more visible and genuinely supportive, it would make a significant difference. The fundamental challenge lies in the limited understanding of the realities we face, which ultimately results in a lack of meaningful support”.*

*(P12)*

The emotional toll of these conditions was evident in participants’ descriptions of deteriorating mental wellbeing:

*“You are constantly stressed. And you’ll end up being aggressive instead of being yourself. You develop depression”.*

*(P8)*

Despite these challenges, nurses remained committed to maintaining therapeutic relationships with patients. Several participants highlighted how emotional presence and attentiveness were integral to patient trust and care quality:

*“Patients seem to sense when you’re genuinely present, and that fosters trust and openness, which enhances their overall healing experience”.*

*(P5)*

Collectively, these accounts illustrate how emotional strain and moral distress are deeply embedded in organizational conditions, shaping nurses’ daily experiences and psychological wellbeing.

##### Sub-Theme: Professional Meaning and Emotional Fulfilment

Alongside emotional strain, participants described moments of deep satisfaction and fulfilment, particularly when patients showed improvement or when dialysis was no longer required. These experiences reinforced nurses’ sense of purpose, resilience and professional identity. Participants expressed fulfilment when patients regained independence from dialysis:

*“When a patient finds a donor, it is deeply rewarding because they no longer have to depend on the dialysis machine”*

*(P7)*

*“We have two patients who have been able to discontinue dialysis due to their improved condition, which is very encouraging,”.*

*(P1)*

*“When a patient’s condition improves and no longer depends on the machine, it gives you strength”.*

*(P2)*

Others emphasized how patient recovery made the emotional demands of the work feel worthwhile:

*“Seeing patients recover makes everything feel worthwhile”.*

*(P6)*

Taken together, these accounts demonstrate that professional meaning and emotional fulfilment coexist with distress. Positive patient outcomes served as powerful sources of motivation and emotional renewal, enabling nurses to sustain their commitment despite ongoing challenges.

#### 3.1.2. Theme: Systemic Resource Constraints

Nurses’ experiences of working in under-resourced settings revealed how systemic limitations shaped their daily practice, ethical decision-making, and the quality of patient care.

##### Sub-Theme: Shortage of Human Resources

Inadequate staffing levels and shortages of nephrology-trained nurses were consistently described by nurses as intensifying workload pressures and compromising the delivery of safe, ethical, and compassionate care. As a result, exhaustion and absenteeism increased, further jeopardizing patient care.

*“There is a shortage of nephrology-trained nurses, which compromises the quality of patient care. As a result, patients do not always receive the standard of care they require, highlighting the need for additional and ongoing training”.*

*(P8)*

Chronic understaffing contributed to exhaustion and absenteeism:

*“When we are overworked, we become exhausted and may be absent from work”.*

*(P9)*

*“We are short-staffed and consistently overworked”.*

*(P4)*

These experiences highlight how workforce shortages undermined nurses’ ability to provide safe, ethical, and compassionate care.

##### Sub-Theme: Material and Infrastructural Shortage

Participants also described persistent shortage of dialysis machines, delayed maintenance, and limited access to essential supplies, all of which disrupted care continuity and intensified emotional strain.

One participant explained:

*“It is overwhelming and emotionally draining because we lose patients due to a lack of resources. Some patients who require dialysis are not treated because the demand exceeds the available supply”.*

*(P11)*

Others reported treatment delays caused by equipment failure:

*“Sometimes patients leave the unit without being dialyzed because the equipment is not working, which means they miss treatment even when there is a clear need”.*

*(P3)*

Overall, material and infrastructural shortages compromised care continuity and contributed to nurses’ frustration, stress, and moral distress.

#### 3.1.3. Recommendations for Practice Improvement

The nurses articulated clear suggestions aimed at strengthening both patient care and nurse wellbeing.

##### Sub-Theme: Staffing and Skill Mix Levels Improvement

The nurses emphasized the need for increased staffing levels and the recruitment of nephrology-trained nurses to reduce workloads and enhance care quality.

One participant noted:

*“Employing additional staff, especially nephrology-trained nurses, would greatly alleviate workload pressures and enhance the quality and continuity of patient care”.*

*(P4)*

Another highlighted the need for both staffing and specialist support:

*“There is also a need for more nephrology nurses, as well as access to psychological support services, such as counselling, a psychologist, or spiritual support from a clergy member”.*

*(P10)*

*“If more nephrologists were trained, it would significantly improve service delivery. There is also a need for the hospital to employ more nurses so that patients on waiting lists can be adequately managed, including during night shifts. Currently, there is a shortage of nephrology-trained nurses, which compromises the quality of patient care, as some staff rely mainly on experience rather than specialized training. This highlights the urgent need for additional and ongoing training”.*

*(P3)*

##### Sub-Theme: Emotional and Professional Support

Participants highlighted the importance of structured emotional support, including counselling and debriefing to help nurses cope with the emotional demands of dialysis care.

*“We would benefit from regular psychological support sessions, perhaps quarterly or twice a year, where psychologists could meet with us to allow space to share our experiences and express our challenges. Losing patients affects us deeply, and having someone who listens and supports us would be helpful”.*

*(P9)*

*“The work is emotionally draining and challenging because you cannot give from an empty cup. Continuous emotional support is needed to help nurses manage these emotional challenges so that they can function better”.*

*(P7)*

##### Sub-Theme: Recognition and Appreciation

Simple acts of recognition were viewed as powerful motivators that could improve morale and performance.

Participants stated that recognition enhanced motivation and performance:

*“Staff appreciation is important because when nurses feel recognized and motivated, it encourages improved performance and commitment to their work”.*

*(P1)*

Others linked appreciation to broader support and development opportunities:

*“There is a need for stronger management support through support groups, as well as more training opportunities, including regular in-service training within the department”.*

*(P2)*

While the findings are presented descriptively, they are interpreted in the discussion through Watson’s Theory of Human Caring to examine how organizational and systemic conditions shape nurses’ capacity to sustain caring-healing practices.

## 4. Discussion

Guided by Watson’s Theory of Human Caring, this discussion interprets nurses lived experiences as relational and ethical practices shaped by organizational and systemic conditions, with implications for care quality and health equity in line with SDG 3 [15]. These findings reflect broader challenges in advancing SDG 3, as inequities in staffing, infrastructure, and emotional support undermine both universal health coverage and the quality of chronic disease care.

### 4.1. Emotional Strain, Moral Distress, and Professional Meaning

The findings indicate that emotional strain and moral distress are central features of dialysis nursing, shaped largely by organizational constraints rather than by patient care itself. To contextualize these findings, comparisons are drawn with previous dialysis nursing studies to highlight both similarities and key differences.

Nurses described persistent emotional exhaustion arising from high workloads, prolonged patient relationships, and repeated exposure to patient suffering, experiences that align with previous qualitative studies conducted in dialysis settings [24,25,26]. Similar to research conducted in other South African and low- and middle-income contexts, participants identified chronic understaffing, limited managerial support, and inadequate resources as key contributors to emotional strain and compromised care quality [27,28]. These parallels suggest that emotional burden in dialysis nursing is a persistent and systemic issue rather than an isolated organizational challenge.

While existing studies largely frame compassion fatigue as the primary consequence of close nurse–patient relationships [29]. This study contributes a more nuanced understanding by demonstrating that emotional strain often coexists with a strong sense of professional meaning and resilience. Unlike earlier research that emphasizes burnout as the dominant outcome of sustained emotional engagement, nurses in this study also described patient recovery, transplantation, and discontinuation of dialysis as powerful sources of fulfilment and professional affirmation. This finding extends existing literature by showing that emotional closeness in dialysis nursing can function simultaneously as a source of emotional strain and emotional renewal, particularly when nurses are able to witness positive patient outcomes.

Interpreted through Watson’s Theory of Human Caring, these findings highlight how nurses’ capacity to sustain authentic presence and transpersonal caring relationships are deeply influenced by organizational conditions. When systemic constraints prevent nurses from practicing in ways aligned with their professional and ethical values, caring labour becomes a source of moral distress rather than professional meaning.

### 4.2. Systemic Resources and Staffing Constraints

Beyond emotional labour, the findings highlight systemic resource and staffing constraints as key determinants of nurses lived experiences and ethical challenges in dialysis care. Resource provision was described as a daily struggle, with shortages of dialysis machines, delayed maintenance, and limited supplies forcing nurses to witness preventable patient suffering. In such circumstances, nurses were often unable to provide timely or adequate care, intensifying moral distress and emotional exhaustion.

These findings reveal a critical tension between nurses’ commitment to holistic, patient-centred care and the structural limitations of the South African public health sector. Operational challenges, including inadequate staffing and malfunctioning equipment, compelled nurses to compromise professional standards, reinforcing feelings of ethical distress. Consistent with existing literature, emotional exhaustion in dialysis nursing emerged as being shaped more strongly by organizational and systemic conditions than by clinical demands alone [18].

This aligns with broader challenges in the South African health system related to human resource management and ethical dilemmas in resource allocation. Previous studies have similarly documented the relationship between inadequate nurse staffing and compromised care quality in haemodialysis units, underscoring the structural nature of these challenges.

### 4.3. Organizational Support, Leadership, and Nurse Wellbeing

Despite structural resource constraints such as inadequate staffing, equipment, and supplies, nurses’ capacity to provide comprehensive care may be undermined. This calls into question the key principles of Watson’s Theory of Human Caring, which emphasizes true presence, empathy, and transpersonal interactions [19]. Nonetheless, Watson’s caring concepts, including emotional support, spiritual comfort, and personalized treatment, have the potential to greatly improve patient experiences and outcomes. Recent research suggests that, even in resource-constrained situations, caring science increases health equity, improves patient adherence, and creates resilience among healthcare personnel, hence improving long-term CKD management in line with SDG 3 goals [15].

The findings further demonstrate that organizational support and leadership practices play a decisive role in shaping nurses’ wellbeing, ethical practice, and ability to sustain caring relationships. Nurses’ reliance on informal coping mechanisms, such as prayer and peer support, reflects a lack of formal institutional psychological support. This absence represents a significant barrier to sustaining quality care, as unmanaged emotional labour contributes to burnout and staff attrition, further straining already under-resourced services.

Although leadership and staffing constraints are well documented in the literature, this study offers deeper insight into how limited managerial presence directly exacerbates nurses’ moral distress. Similar patterns have been reported in South African nursing research, where inadequate leadership engagement is associated with ethical tension and reduced job satisfaction [30]. Viewed through Watson’s Theory of Human Caring, the absence of visible and supportive leadership constrains nurses’ ability to sustain caring-healing environments, authentic presence, and compassionate engagement, highlighting that organizational shortcomings affect not only operational efficiency but also the ethical and relational core of nursing practice.

Participants also emphasized the importance of recognition and appreciation. Simple acts of acknowledgment such as verbal encouragement or recognition of effort were perceived as powerful motivators that could restore dignity and morale in environments where nurses often felt unseen. Cultivating a culture of appreciation thus emerges as a low-cost but meaningful strategy for sustaining wellbeing in nursing.

### 4.4. Health Equity, International Comparisons, and SDG 3

These findings must be understood within the broader health equity agenda articulated in SDG 3, which emphasizes universal health coverage, quality healthcare services, and the reduction in non-communicable disease burden. Nurses’ struggles to provide high-quality, equitable dialysis care reflect systemic barriers to achieving these goals within the public health sector.

Notable differences emerge when these findings are compared with studies conducted in better-resourced dialysis settings. Research from high-income countries highlights the availability of structured psychological support, formal debriefing mechanisms, and specialist staffing models that buffer emotional strain and sustain nurse wellbeing [31]. In contrast, nurses in the present study relied largely on informal coping strategies, such as peer support and spirituality, reflecting limited institutional provision for emotional support. This contrast underscores how broader health system inequities shape nurses’ lived experiences and reinforces the importance of interpreting dialysis nursing within resource-constrained public-sector contexts.

By framing nurses’ emotional and relational experiences within Watson’s Theory of Human Caring, this study provides a theoretical basis for advocating interventions that support nurse wellbeing as a prerequisite for improving patient outcomes. Strengthening organizational support, staffing, and emotional care for nurses is therefore integral to advancing equitable, high-quality CKD services in alignment with SDG 3 [15].

## 5. Study Limitations

- This study has several limitations that should be considered when interpreting the findings. First, the research was conducted in a single dialysis unit within an academic hospital in Tshwane, which may limit the transferability of the findings to other dialysis settings. Although rich, in-depth data were generated, the experiences captured reflect a specific organizational and resource environment.
- Second, our study only included female professional nurses. We missed out on the different perspectives that male colleagues or other team members, like managers and enrolled nurses, might have shared. While this group represents the heart of our unit, a broader mix of voices would have given us a more complete picture of how the whole team experiences the challenges of renal care.
- Third, since the data relied on personal accounts shared within the workplace, some participants might have held back or remembered things differently to maintain a professional image. Even with our focus on creating a safe and private space, the natural pressure of the environment may have influenced how openly they felt they could speak.
- Finally, while Watson’s Theory helped us focus deeply on the emotional and human side of nursing, looking through just one lens means we might have paid less attention to bigger issues like workplace policies or organizational structure. In the future, studies could adopt multi-theoretical or mixed methods approaches to strengthen analytic depth and generalizability. These limitations suggest caution when applying findings to differently resourced dialysis units.

## 6. Recommendations

- Strengthening dialysis care depends on adequate nurse staffing, particularly the recruitment and retention of nephrology-trained nurses, as well as meaningful emotional support and ongoing professional development.
- Visible and supportive leadership plays a key role in sustaining morale, ethical practice, and compassionate care.
- At a policy level, prioritizing renal services in workforce planning, ensuring reliable dialysis equipment, and recognizing nurse wellbeing as a quality indicator are essential.
- Addressing both the human and systemic dimensions of care is critical for supporting dialysis nurses, protecting patient outcomes, and strengthening the long-term sustainability of CKD services in line with SDG 3.

## 7. Conclusions

This study highlights the complex and emotionally charged environment in which dialysis nurses operate within a resource-constrained public health setting. Interpreted through Watson’s Theory of Human Caring, the findings demonstrate that nurses’ experiences of moral distress and professional fulfilment are shaped by their ability or inability to sustain authentic presence, transpersonal relationships, and caring-healing environments. Addressing these conditions is essential for advancing equitable, high-quality CKD care in alignment with SDG 3.

Despite their dedication, nurses face persistent challenges, including resource shortages, high workloads, inconsistent managerial support, and limited opportunities for professional development. These systemic barriers compromise both patient outcomes and nurse wellbeing. As CKD prevalence continues to rise, strengthening dialysis nursing is essential for sustainable and equitable kidney care.

## Figures and Tables

**Table 1:** Demographic Characteristics of Participating Professional Nurses (N = 12).

Participant ID	Age (Years)	Highest Professional Qualification	Specialist Qualification in Nephrology	Years of Nursing Experience	Years of Experience in Renal Unit
1	34	Diploma (GPMC) and Midwife	No	9	4
2	43	Diploma (GPMC) and Midwife	No	9	6
3	43	Bachelor’s Degree (GPMC) and Midwife	Yes	19	19
4	48	General Nurse (Bridging)	No	17	17
5	45	Diploma (GPMC) and Midwife	Yes	18	18
6	55	Bachelor’s Degree (GPMC) and Midwife	Yes	22	9
7	46	Diploma (GPMC) and Midwife	No	18	1.1 (1 year 1 month)
8	49	Diploma (GPMC) and Midwife	No	7	1.25 (1 year 3 months)
9	50	Diploma (GPMC) and Midwife	Yes	10	10
10	43	Bachelor’s Degree (GPMC) and Midwife	No	19	3
11	52	Diploma in General Nursing and Midwife	Yes	16	16
12	42	Bachelor’s Degree (GPMC) and Midwife	Yes	12	12

**Table 2:** Experiences of Nurses Caring for Patients with Chronic Kidney Disease.

Themes	Sub-Themes
Emotional and Professional Experiences	Emotional strain and moral distress
	Professional meaning and emotional fulfilment
Systemic Resource Constraints	Shortage of human resources
	Material and infrastructural shortages
Recommendations for Practice Improvement	Staffing and skill mix
	Emotional and professional support Recognition and appreciation

## Data Availability

The data presented in this study are not publicly available due to privacy and ethical restrictions to protect participant confidentiality.

## Abbreviations

The following abbreviations are used in this manuscript:

AKI	Acute Kidney Injury
CKD	Chronic Kidney Disease
HD	Haemodialysis
PD	Peritoneal Dialysis
SDG	Sustainable Development Goals
